# Controlled Attenuation Parameter for Quantification of Steatosis: Which Cut-Offs to Use?

**DOI:** 10.1155/2021/6662760

**Published:** 2021-03-26

**Authors:** Roxana Sirli, Ioan Sporea

**Affiliations:** Department of Gastroenterology and Hepatology, Advanced Regional Research Center in Gastroenterology and Hepatology, “Victor Babeş” University of Medicine and Pharmacy, 156, Liviu Rebreanu Bv., Timişoara 300723, Romania

## Abstract

Chronic liver diseases (CLDs) are a public health problem, even if frequently they are underdiagnosed. Hepatic steatosis (HS), encountered not only in nonalcoholic fatty liver disease (NAFLD) but also in chronic viral hepatitis, alcoholic liver disease, etc., plays an important role in fibrosis progression, regardless of CLD etiology; thus, detection and quantification of HS are imperative. Controlled attenuation parameter (CAP) feature, implemented in the FibroScan® device, measures the attenuation of the US beam as it passes through the liver. It is a noninvasive technique, feasible and well accepted by patients, with lower costs than other diagnostic techniques, with acceptable accuracy for HS quantification. Multiple studies have been published regarding CAP performance to quantify steatosis, but due to the heterogeneity of CLD etiologies, of steatosis prevalence, etc., it had widely variable calculated cut-off values, which in turn limited the day-to-day utility of CAP measurements in clinical practice. This paper reviews published studies trying to suggest cut-off values usable in clinical practice.

## 1. Introduction

Chronic liver diseases (CLDs) are a public health problem, even if frequently they are underdiagnosed. A study from 2014 estimated that 844 million individuals are affected by CLD, with a mortality rate of 2 million per year [1]. The most frequent CLDs are chronic viral hepatitis, alcoholic liver disease (ALD), and nonalcoholic fatty liver disease (NAFLD) with its progressive variant-nonalcoholic steatohepatitis (NASH). Even if effective treatments are available for chronic viral hepatitis, in NAFLD and NASH this is not the case, an alarming fact considering that the world-vide pooled prevalence of NAFLD is estimated to be 25.24% [2], ranging from approximately 13% in Africa to approximately 30% in Asia and South America. Furthermore, the prevalence of NAFLD is expected to increase since the prevalence of its etiologic factors (obesity, diabetes mellitus, hypertriglyceridemia) is increasing.

Hepatic steatosis (HS) is encountered not only in NAFLD, but also in chronic viral hepatitis, alcoholic liver disease, etc. Several studies demonstrated that HS plays an important role in fibrosis progression, regardless of CLD etiology [3, 4], and that it impairs response to treatment in chronic viral hepatitis [5].

## 2. Diagnosis of Hepatic Steatosis

Considering all these facts, detection and quantification of HS are imperative, but also a challenge. Detection of HS relies mainly on imaging methods. B-mode ultrasonography is usually the first-line imaging method to detect HS, but it cannot assess the presence of inflammation and it is imprecise to assess steatosis severity, especially mild [6, 7]. Magnetic resonance imaging (MRI) techniques, especially proton density fat fraction (PDFF), are very accurate to detect and quantify HS [8], but they are very expensive and not available enough to be used for assessment of such a large number of patients.

Liver biopsy is considered the gold standard for assessing HS severity, as well as inflammation and fibrosis, when they are present [6, 7]. According to histologic findings, liver steatosis is classified as absent-*S*_0_ (normal liver), when less than 5% of the hepatocytes have fatty infiltration; mild-*S*_1_, when 5 up to 33% of the hepatocytes present fatty infiltration; moderate-*S*_2_, 33–66% of the hepatocytes with fatty infiltration; and severe-*S*_3_, more than 66% of the hepatocytes with fatty infiltration [6, 7]. However, liver biopsy is an invasive method, poorly accepted by the patients, especially if repetitive, and there are some problems regarding inter-observer variability in assessing the sample, as well as regarding sampling errors [9]. Furthermore, the applicability of liver biopsy to assess such a huge number of patients is highly questionable.

Considering all these facts, noninvasive methods have been developed to assess HS, as well as inflammation and fibrosis (when present). They include biomarkers and imaging techniques [6, 7]. Among the imaging techniques, the controlled attenuation parameter (CAP) feature, implemented on the FibroScan® device, seems the most promising noninvasive test to quantify HS.

## 3. Controlled Attenuation Parameter (CAP): Technical Data

Vibration-controlled transient elastography (VCTE) (FibroScan®, EchoSens, Paris, France) is an ultrasound-based elastography technique developed more than 15 years ago, firstly used for fibrosis assessment in chronic liver diseases. It is the most validated elastography technique, accepted by international guidelines as a reliable tool to quantify liver fibrosis [10, 11]. VCTE measures the velocity of shear waves generated inside the liver by a mechanical impulse. In CLD, liver stiffness increases with the progression of fibrosis. The stiffer the liver is, the higher the shear waves' velocity.

Several years later, CAP feature was added to the FibroScan® device. It measures the attenuation of the US beam as it passes through the liver. CAP correlates with the viscoelastic characteristics of the liver, dependent in their turn on the quantity of fat droplets in the hepatocytes [12]. CAP measurements can be performed by either the M or XL probes (chosen according to the skin to liver capsule distance), and the results are expressed in decibels per meter (dB/m), ranging from 100 to 400 dB/m [13]. At the beginning, CAP was available only on the M probe of the FibroScan®. Later, it was implemented also on the XL probe developed for obese subjects.

The initial studies regarding CAP showed excellent feasibility-92.3% of cases with only the M probe [13], improved to 96.8% when both M and XL probes have been used [14], also with excellent reproducibility, inter-rater agreement 0.82–0.84 with the M probe [15, 16], but lower with the XL probe, 0.75 and 0.65, respectively [14, 15].

No quality technical parameters have been recommended by the producers to ensure reliable measurements. Therefore, most authors used the quality criteria recommended for VCTE: 10 valid measurements with an IQR/M < 30% [17, 18]. A study published in 2017 recommended as a quality criterion for CAP measurements an IQR < 40 dB/m [19]. When this quality criterion was used, the AUROC of CAP to assess steatosis as compared to liver biopsy increased from 0.77 to 0.9. Another study has set the IQR upper limit at 30 dB/m [8], while another study found no difference in CAP performance when the IQR was ≥30 dB/m or ≥40 dB/m [20]. A recently published study demonstrated that CAP-IQR/M < 0.3 as a quality criterion improves accuracy and feasibility of CAP measurements, performing better than the IQR < 40 dB/m criterion [21].

Regarding the use of M vs. XL probe to assess steatosis grade by CAP, data is still conflicting. In a study performed in a Caucasian population, the cut-off and performance were similar for M vs. XL probe [22], while in a smaller study performed in a Chinese population, cut-off values were higher with the XL probe, but the performance was similar [23]. In a very recent study in Japanese population, cut-off values were higher for the XL probe, but there were no significant differences in accuracy [24].

Several studies demonstrated that CAP measurements are not influenced by the severity of liver fibrosis, nor by the presence of cirrhosis [25–28]. However, several factors have been proven to influence CAP values, among them BMI [29, 30], the presence of diabetes and etiology, especially NAFLD [29], while CAP values higher than 300 dB/m may lead to an overestimation of fibrosis severity by VCTE in patients with lower stages of fibrosis [31].

## 4. Controlled Attenuation Parameter (CAP): Predictive Value for Steatosis Severity in Individual Studies

Up to date, numerous studies have been published regarding the predictive value of CAP for steatosis severity. We summarized in Table 1 data from studies including more than 100 subjects, with liver biopsy as the reference method, CAP measurements being performed with the M probe (Table 1).

As it can be seen, the performance of CAP for detecting any steatosis (*S* ≥ 1) is very good, the AUROC usually being higher than 0.8. In populations with mixed etiology of CLD, the AUROCs remain also high for diagnosing more severe steatosis (*S*_2_ and *S*_3_). However, in NAFLD population, the AUROCs for diagnosing moderate (*S*_2_) and severe (*S*_3_) steatosis decrease, sometimes as low as 0.58 [39], or even 0.37 [38]. Nevertheless, the severity of fat infiltration in NAFLD does not affect prognosis [45], so the important thing is to detect even mild steatosis (*S*_1_), for which CAP is much better than B-mode ultrasonography [46].

The largest individual study assessing the value of CAP for predicting fibrosis severity was published in 2019 by Eddowes et al. [20]. It was a multicenter prospective study that included 450 patients with NAFLD evaluated by CAP/TE and liver biopsy. The AUROCs of CAP to identify patients' steatosis were as follows: for *S* ≥ *S*_1_-AUROC of 0.87; for *S* ≥ *S*_2_-0.77; while for *S*_3_ it was 0.70. Youden cut-off values were 302 dB/m for *S* ≥ *S*_1_, 331 dB/m for *S* ≥ *S*_2_, and 337 dB/m for *S*_3_.

The cut-offs also vary a lot among the studies. An explanation could be the relatively small number of patients included in each study, the heterogeneity among groups regarding etiology, overall steatosis prevalence, and also among steatosis severity groups.

To overcome these shortcomings, meta-analyses have been performed.

## 5. Controlled Attenuation Parameter (CAP): Predictive Value for Steatosis Severity in Meta-Analyses

The first published meta-analysis included nine studies with 11 cohorts, totalizing 1771 patients with CLD of diverse etiologies [47]. The summary sensitivities and specificities values were 0.78 and 0.79 for *S* ≥ 1; 0.85 and 0.79 for *S* ≥ 2; 0.83 and 0.79 for *S*_3_, respectively. The HSROCs were 0.85 for *S* ≥ 1, 0.88 for *S* ≥ 2, and 0.87 for *S*_3_. The median optimal cut-off values of CAP for *S* ≥ 1, *S* ≥ 2, and *S*_3_ were 232.5 dB/m (range 214–289 dB/m), 255 dB/m (range 233–311 dB/m), and 290 dB/m (range 266–318 dB/m).

The second meta-analysis included 11 studies with 13 cohorts, all of them with high methodological quality, totalizing 2076 patients with CLD of diverse etiologies [48]. The summary sensitivity, specificity, and AUC for *S* ≥ 1 were 0.78, 0.79, and 0.86, respectively; for *S* ≥ 2, they were 0.82, 0.79, and 0.88, respectively, while for *S*_3_ they were 0.86, 0.89, and 0.94, respectively. Significant heterogeneity was found among the studies for *S* ≥ 1 and *S*_3_. CAP cut-of values for *S* ≥ 1 ranged from 214 to 289 dB/m, median 238 dB/m; for *S* ≥ 2 they ranged from 230 to 311 dB/m, median 259 dB/m, while for *S*_3_ CAP values ranged from 266 to 327 dB/m, median 290 dB/m.

Both meta-analyses above were not able to provide optimized cut-offs with high predictive values due to the limitations of conventional meta-analyses and to the heterogeneity of the included studies, so that a third meta-analysis was performed, this time using individual patient data from 19 studies, including 2735 CLD cases of various etiology, with liver biopsy and CAP measurements [29]. The overall performance of CAP in this meta-analysis was as follows: for *S* ≥ 1 the calculated cut-off was 248 dB/m, with 0.68 sensitivity and 0.82 specificity (AUROC 0.82); for *S* ≥ 2, the calculated cut-off was 268 dB/m, with 0.77 sensitivity and 0.81 specificity (AUROC 0.86), while for *S*_3_ the calculated cut-off was 280 dB/m, with 0.88 sensitivity and 0.77 specificity (AUROC 0.88).

Another important finding of this last meta-analysis is the fact that, among etiologies, only NAFLD seems to influence CAP values. In other words, NAFLD patients have higher CAP values (by 10 dB/m) as compared with all other etiologies of CLD for the same grade of histologic steatosis [29]. Furthermore, it was calculated that BMI, as well as the presence of diabetes mellitus, influences CAP values. Considering these findings, the authors propose an algorithm to correct the measured CAP values, and to apply the cut-offs only after the corrections are made. These correction include deducting 10 dB/m for the presence of NAFLD/NASH, as well as for diabetes mellitus, deducting 4.4 dB/m for each BMI unit over 25 kg/m^2^, or adding 4.4 dB/m for each BMI unit bellow 25 kg/m^2^.

Finally, a recently published meta-analysis assessed only NAFLD patients (1297 subjects) evaluated by liver biopsy and CAP in nine studies [49]. The mean AUROC, pooled sensitivity, and pooled specificity for diagnosing *S* ≥ 1 were 0.96, 0.87, and 0.91, respectively; for *S* ≥ 2, they were 0.82, 0.85, and 0.74, respectively, while for *S*_3_ they were 0.70, 0.76, and 0.58, respectively. As observed in individual studies (Table 1), in NAFLD patients the performance of CAP to diagnose steatosis severity decreases as the steatosis progresses. No polled cut-off values have been calculated in this meta-analysis.

## 6. Controlled Attenuation Parameter, Transient Elastography, and NAFLD/NASH

As mentioned before, the prevalence of NAFLD/NASH is increasing worldwide and in the future will be the main cause of liver-related morbidity and mortality. Considering the high number of patients and the fact that not all patients with NAFLD will develop NASH and liver related events, it is not feasible to try to evaluate all of them by liver biopsy, thus the utility of noninvasive methods. As shown before, individual studies [20, 24, 30, 32, 35–39] and meta-analyses [49] proved the value of CAP for diagnosing steatosis in patients with NAFLD/NASH, even if accuracy decreases with the severity of steatosis [49].

VCTE is the most validated elastographic method for fibrosis assessment in NAFLD/NASH. The cut-off values for different stages of fibrosis vary according to the probe used. For the XL probe (developed especially for obese patients), the cut-offs are as follows: 6.2 kPa for *F* ≥ 2, 7.2 kPa for *F* ≥ 3, and 7.9 kPa for *F*_4_ [50]. For the M probe the cut-offs are as follows: 7 kPa for *F* ≥ 2, 8.7 kPa for *F* ≥ 3, and 10.3 kPa for *F*_4_ [51]. In a recent meta-analysis that included 854 NAFLD patients from eight studies, TE had 79% Se and 75% Sp for diagnosing *F* ≥ 2 and 85% Se and Sp for diagnosing *F* ≥ 3, while for cirrhosis the Se and Sp were 92% [52]. No cut-offs were provided. The accuracy of TE increases with the severity of fibrosis; thus, TE is a very good method to rule in and to rule out cirrhosis.

## 7. Final Considerations

The ideal diagnostic test should be accurate, available, noninvasive, feasible, inexpensive, and acceptable by the patient. All the data that we presented above suggest that CAP is a feasible test with good accuracy for the detection and quantification of hepatic steatosis, if clinical aspects, such as BMI and presence of diabetes mellitus and of NAFLD/NASH, are taken into consideration. Regarding availability, FibroScan® device is readily available in European countries such as France and even Romania, and, a few years ago, FDA accepted it as a valuable tool to assess fibrosis in the United States. Since it is noninvasive, and it takes only a few minutes to perform, VCTE and CAP are well accepted by the patients. Thus, in some countries, VCTE and serologic markers replaced almost entirely liver biopsy for fibrosis severity assessment [53]. Regarding CAP costs, they are included in those of VCTE assessment of fibrosis and are much lower than of PDFF-MRI, even if with a small loss of accuracy.

Considering all of the above, the rise in NAFLD/NASH prevalence, as well as the steatosis impact on the prognosis of CLD, CAP could be used as a screening tool in patients at risk for NAFLD/NASH (diabetics, obese, patients with metabolic syndrome). Regarding cut-offs to be used, those calculated by the Karlas meta-analysis seem the most robust since they were calculated starting from a large individual data-base meta-analysis and since they take into consideration factors known to influence CAP measurements [29].

The main advantages and weaknesses of CAP/VCTE are summarized in Table 2.

## 8. Conclusion

Controlled attenuation parameter is a valuable tool to detect hepatic steatosis in day-to-day clinical practice. Cut-off values of 248 dB/m, 268 dB/m, and 280 dB/m, corrected by BMI and presence of co-morbidities, can be taken into consideration to diagnose *S* ≥ 1, *S* ≥ 2, and *S*_3_.

## Figures and Tables

**Table 1:** Performance of CAP (M probe) to diagnose steatosis in patients with CLD, with liver biopsy as the reference method.

Author	No. of patients	Etiology	Prevalence of *S* ≥ 1 (%)	*S* ≥ 1		*S* ≥ 2		*S* = 3	
				Cut-off (dB/m)	AUROC	Cut-off (dB/m)	AUROC	Cut-off (dB/m)	AUROC
Sasso [28]	615	HCV	30	222	0.80	233	0.86	290	0.88
Myers [27]	153	Mixed	65	283	0.81	—	—	—	—
De Ledinghen [25]	112	Mixed	48	215	0.84	252	0.86	296	0.93
Chan [32]	105	NAFLD	97	263	0.97	281	0.86	283	0.75
De Ledinghen [13]	440	Mixed	51.5	—	0.79	—	0.84	—	0.84
Ferraioli [26]	114	Mixed	42.6	219	0.76	296	0.82	—	—
Lupsor-Platon [33]	201	Mixed	45.3	260	0.81	285	0.82	194	0.84
Shen [34]	332	Mixed	42.5	255	0.88	283.5	0.90	293.5	0.84
De Ledinghen [35]	261	NAFLD	100	—	—	310	0.80	311	0.66
Imajo [36]	142 (10 controls)	NAFLD	83	236	0.88	279	0.73	302	0.70
Park [37]	104	NAFLD	91	261	0.85	305	0.70	312	0.73
Naveau [38]	123	NAFLD	81	298	0.81	303	0.58	326	0.37
Siddiqui [39]	393	NAFLD	95	285	0.76	311	0.70	306	0.58
Thiele [40]	269	Alcoholic liver disease	72	290-rule-in 220-rule-out	0.77	328-rule-in 257-rule-out	0.78	339-rule-in 286-rule-out	0.83
Shalimar [30]	219	NAFLD	93.2	285	0.96	331	0.71	348	0.75
Oeda [24]	137	NAFLD	96.3	—	—	264	0.64	289	0.69
Somda [41]	249	Severely obese	84.3	255	0.86	288	0.83	297	0.79
Eddowes [20]	450	NAFLD	88	302	0.87	331	0.77	337	0.70
Baumeler [42]	224	Mixed	62.1	258.5	0.78	282.5	0.83	307.5	0.82
Trowell [43]	217	Mixed	43	278	0.82	301	0.79	—	—
Zeng [44]	173	Liver donors	—	244	0.88	—	0.89	—	—

CAP: controlled attenuation parameter; S: steatosis; AUROC: area under the receiver operating characteristic curve; HCV: hepatitis C virus; NAFLD: nonalcoholic fatty liver disease.

**Table 2:** Main advantages and weaknesses of CAP/VCTE.

Advantages	Weaknesses
(i) Reproducible method	(i) Expensive equipment
(ii) Well accepted by the patients and thus repeatable assessment possible for follow-up	(ii) Not feasible in patients with ascites
(iii) Good results for noninvasive steatosis assessment in patients with CLD, including NASH	(iii) Increased number of unreliable measurements in patients with high BMI, especially with M probe
(iv) CAP could be used as a screening tool in patients at risk for NAFLD/NASH	(iv) CAP not very accurate to differentiate *S* ≥ 2 from *S*_3_
(v) Real-time assessment not only of steatosis but also of fibrosis severity	(v) TE not very accurate to differentiate patients without fibrosis and those with mild fibrosis and patients with moderate vs. mild fibrosis
(vi) Reliable tool for noninvasive assessment of fibrosis, recognized by international guidelines	—
(vii) Results and technical parameters IQR/M available in real time, automatically calculated by the device's software	—

CAP: controlled attenuation parameter; VCTE: vibration-controlled transient elastography.
